# Characteristics of herring marinated in reused brines after microfiltration

**DOI:** 10.1007/s13197-018-3343-3

**Published:** 2018-09-15

**Authors:** Mariusz Szymczak, Katarzyna Felisiak, Barbara Szymczak

**Affiliations:** 1 Department of Food Science and Technology, Faculty of Food Science and Fisheries, West Pomeranian University of Technology, Papieża Pawła VI 3 str., 41-459 Szczecin, Poland; 2 Department of Microbiology and Applied Biotechnology, Faculty of Food Science and Fisheries, West Pomeranian University of Technology, Papieża Pawła VI 3 str., 41-459 Szczecin, Poland

**Keywords:** Herring, Marinating, Brine waste, By-products, Filtration

## Abstract

Brines after herring marinating pose a serious financial problem to the industry and natural environment. Paradoxically, the brine waste containing biological active compounds like proteases and peptides being responsible for marinade quality is discarded with sewage. Results show that the reuse of brine without filtration is not possible because of increase in the bacterial count and lipid oxidation in marinated herring. The desired parameters of marinades were achieved using brine permeate-50 µm free of the suspension and lipids. The best quality and sensory parameters of marinated herring meat were obtained using permeate-0.22 brine, which not contain microorganisms and lipids, and also a high activity of proteases. Reuse of brine allows reducing by half the losses of protein hydrolysis products (PHP) from meat to brine and for reverse diffusion of PHP and peptidases to meat. The marinades produced with the newly-developed method had up to 25% more PHP, up to 20% lower hardness, 10–20% higher activity of proteases, 40–97% lower indices of lipid oxidation, and 5% higher scores in sensory assessment, compared to the marinades produced with fresh brine. The inexpensive and easy to perform microfiltration of brine affords the possibility of increasing the quality and nutritional value of marinades and minimizing both waste volume and production costs.

## Introduction

Fish marinades that are popular in many regions of the world are manufactured during cold ripening of fish meat in an aqueous solution of table salt, NaCl (4–9%) and acetic acid (3–7%) called a marinating brine. Depending on the herring (Atlantic vs. Baltic), fat content in fish, season and basic parameters (fish to brine ratio, temperature, marinating time) different concentrations of salt and acetic acid are used in the world.

The ripening of marinade meat proceeds as a result of multiple physical, biochemical, and microbiological transformations. Most of these transformations are associated with the hydrolysis of proteins and lipids and with their interactions. In marinades with low pH values, active are acidic aspartyl proteases like cathepsin D and E and pepsin, as well as cysteine proteases like cathepsin B and L (Szymczak 2017; Szymczak and Lepczyński 2016). Within the first 100 h of the marinating process, the fastest increase is observed in the content of peptides which is due to the activity of endopeptidases, while after 5–7 days increases may be noticed in the contents of free amino acids as a result of the activity of exopeptidases (Szymczak 2017). Protein hydrolysis products, especially these with a low molecular weight, are responsible for the characteristic sensory traits of marinated meat (Nielsen et al. 2004). The significant loosening of meat structure is also caused by dissolution of poorly crosslinked collagen in acetic acid (Szymczak et al. 2015).

Today, the quality of marinades produced on the industrial scale is unsatisfactory because producers use: very short time of marinating (3–4 days), mainly frozen-thawed raw material, low temperature (1–4 °C), and high concentrations of salt and acetic acid (above 7%). The best quality marinades are obtained using the traditional parameters used in these studies. In addition, nitrogen fractions which impart the characteristic taste to meat of marinades diffuse from meat to the brine (Szymczak and Kołakowski 2012).

Once the fish marinating process is completed, the brine is discarded with sewage. Due to the high content of protein and lipids, the brine is pre-purified using physical and chemical methods. This results in both contamination of the natural environment and economic losses. The cost of producing brine and its utilization is as much as 10% of the product price of marinades. The recovery of only salt and acetic acid from the brine is cost-ineffective and may only be driven by environment protection concerns, while the brine contains more valuable biologically active substances. Investigations conducted by Albertos et al. (2016) and by Gringer et al. (2016) indicate that brine peptides exhibit a high antioxidative activity. They may protect fish lipids against oxidation, while in marinades a similar effect may be achieved through the addition of plant extracts (Topuz et al. 2014).

Recent researches have shown that, despite low pH value and high concentration of salt, the marinating brine contains active aspartyl and cysteine cathepsins (Szymczak 2017; Szymczak and Lepczyński 2016). The analytic preparations of cathepsins available in retail are expensive which makes their use in the fish processing industry impossible. From the technological and consumer perspective, especially important should be to increase the role of enzymes and bioactive peptides in the ripening process of fish meat, as this may increase its sensory and nutritive values. Therefore, there is a need to demonstrate if brine waste, which contains high-value marine biomolecules such as enzymes, peptides and free amino acids, could be re-utilized and valorized. The use of the brine waste may, however, be impaired by potential microbiological and chemical hazards. This problem may be eliminated by the purification of cathepsins recovered from the marinating brine (Szymczak 2016b). But even a less expensive and easier way would be to use these enzymes as a partially-purified brine.

Previous attempts have been made to regenerate brine through high-temperature treatment. Although this method reduces microbiological contamination of the brine, it has adverse effects on its lipids and enzymes. Heat treatment, centrifugation, drum drying or evaporation are cost-ineffective in brine waste purification. Chemical methods, like the use of hydrogen peroxide, are forbidden and unsuitable for the recovery of bioactive substances. Today, brine purification is highly effective when conducted with pressure membrane process (Nędzarek et al. 2017). In turn, the most popular method—ultrafiltration—has been shown to reduce cathepsins activity in treated surimi waste due to excessive temperature and fouling (DeWitt et al. 2002).

So far, there have been no works demonstrating the effect of reuse of the brine with active cathepsins and peptides on the marinating process of herrings. The extent of peptides and proteases diffusion from brine to meat and the effect of this diffusion on the quality of marinated meat are unknown either. Hence, the objective of this study was to determine properties of herring meat marinated in the brine left after the earlier marinating process and purified via microfiltration.

## Materials and methods

### Herring

Atlantic herring (*Clupea harengus harengus* L.) was purchased as frozen fillets (size 4–8) in blocks weighing ca. 20 kg, packed in plastic bags stored at − 18 ± 1 °C. Fillets had a length of 17.0 ± 2.8 cm and weighed 142.8 ± 3.1 g; their physicochemical characteristics have been shown in Table 1. Prior to marinating, the frozen fish was thawed up to 2–4 °C in a container with continuous circulation of water at 10 ± 2 °C.

**Table 1:** Characteristics of herring fillets used for marinating

Assay	Unit	Frozen-thawed herring fillet meat
NaCl	%	0.35 ± 0.06
Acetic acid		0.34 ± 0.01
Moisture content		65.1 ± 0.40
pH	Value	6.54 ± 0.03
Total N	g·100 g^−1^	2.35 ± 0.15
Non-protein N	mg·100 g^−1^	251 ± 5
PHB(R)		40.5 ± 3
PHB(A)		6.65 ± 0.7
α-amine N		31.7 ± 0
N-TVB	mg·100 g^−1^	10.4 ± 0.4
N-TMA		0.05 ± 0.08
N-NH3		10.4 ± 0.4
Lipids	%	18.7 ± 0.30
AsV	Value·100 g^−1^ lipids	4.17 ± 0.06
PV	mEq O_2_·kg^−1^ lipids	7.62 ± 0.03
Totox	Value	19.4 ± 0.10
GPA	U_Hb_	22.8 ± 2.1
Cat D	%	82.9
Aspartyl peptidases	U_MCA_	0.5 ± 0.03
Cysteine peptidases		0.1 ± 0.01
TEAC-H_2_O	µTE·g^−1^	0.94 ± 0.22
TEAC-MeOH		2.34 ± 0.07
RSA		0.127 ± 0.002
FRAP		51.3 ± 3.10
Psychrophiles	Log(cfu·g^−1^)	1.65
Mesophiles		0
Molds and yeast		< 1
LAB		< 1

For details see “Materials and methods” section

### Brine

The herrings were marinated in the brine waste originating from the near-by fish processing plant and immediately transported in plastic (polypropylene) buckets with cover (10 l volume) to the laboratory under cold storage conditions. Brine waste was used in the marinating process as: (1) unfiltered brine containing suspended matter and lipids (UB), (2) brine permeate obtained using a 50 µm filter (BP50µ), and (3) brine permeate obtained using a 0.22 µm filter (BP022µ). Microfiltration was conducted using filters made of glass fiber (ø 47 mm) mounted in a vacuum filtration unit (type 162, Sartorius AG, Gottingen, Germany). The filter was exchanged after filtrating 100–150 mL of the brine. The characteristics of the unfiltered brine waste (UB) and brine permeates has been presented in Table 2. Next, salt and acetic acid concentrations were refilled in the UB and permeates up to 6 and 5%, respectively. All stages of the purification process were conducted at a temperature of 5 ± 1 °C using sterile materials. Marinades ripening in the fresh brine (FB) with the final concentration of NaCl and acetic acid at 6 and 5%, respectively, served as the control sample. The brine was prepared from tap water, rock salt and 80% vinegar essence.

**Table 2:** Characteristic of brines waste used for herring marinating process before refilling salt and acetic acid concentration

Analysis/parameter	Unit	Unfiltered Brine (UB)	Brine permeate	
			BP50µ	BP022µ
NaCl	%	3.82 0.03^a^	3.78 0.02^a^	3.77 0.02^a^
Acetic acid		1.30 ± 0.02^a^	1.30 ± 0.02^a^	1.31 ± 0.02^a^
pH	Value	4.481 ± 0.001	4.475 ± 0.001^a^	4.478 ± 0.001^a^
Total N	mg·100 ml^−1^	775 ± 2^a^	751 ± 4^a^	701 ± 1
Non-protein N		475 ± 0^a^	472 ± 4^a^	474 ± 0^a^
PHB(R)		255 ± 5^a^	247 ± 4^a^	260 ± 8^a^
PHB(A)		54 ± 2^a^	55 ± 2^a^	54 ± 4^a^
α-amine N		92 ± 0^a^	95 ± 0	92 ± 0^a^
N-TVB	mg·100 ml^−1^	9.60 ± 0.10^a^	9.50 ± 0.20^ab^	9.10 ± 0.10^b^
N-TMA		0.85 ± 0.05^a^	0.99 ± 0.06^a^	0.94 ± 0.08^a^
N-NH_3_		8.72 ± 0.08^a^	8.48 ± 0.21^ab^	8.11 ± 0.14^b^
Lipids	%	0.43 ± 0.02	0.01 ± 0.003	0
LA	Value·100 g^−1^ lipids	17.7 ± 1.30	41.6 ± 24	–
PV	mEq O_2_·kg^−1^ lipids	75.5 ± 1.80	129 ± 12	–
Totox	Value	168.7 ± 4.0	299 ± 36	–
GPA	U_Hb_	53.0 ± 3.2^a^	39.8 ± 2.0	48.4 ± 1.5^a^
Cat D	%	90	87	89
Aspartyl peptidases	U_MCA_	0.8 ± 0.05^a^	0.6 ± 0.04	0.86 ± 0.07^a^
Cysteine peptidases		0.2 ± 0.01^a^	0.15 ± 0.01^b^	0.22 ± 0.01^ab^
TEAC-H_2_O	µTE·ml^−1^	1.13 ± 0.10^a^	1.09 ± 0.02^a^	1.12 ± 0.01^a^
TEAC-MeOH		2.37 ± 0.11^a^	2.35 ± 0.11^a^	2.01 ± 0.11^a^
RSA		0.135 ± 0.001	0.126 ± 0.003	0.118 ± 0.002
FRAP		77.3 ± 4.1^a^	76.6 ± 7.5^a^	71.1 ± 3.2^a^
Psychrophiles	Log(cfu·ml^−1^)	3.08	3.00	0
Mesophiles		1.90^a^	1.85^a^	1.70
Molds and yeast		2.30	0	0
LAB		2.30^a^	2.08^a^	0

(–) not analyzed

^abc^Means in row without lower case letter or with different lower case letter differ significantly (*P* < 0.05); For details see “Materials and methods” section

### Marinating process

Frozen-thawed herring (1125 ± 1 g) was placed in 2 dm^3^ glass jars that were filled with brine. The marinating process was done in three replications at 7 °C for any kind of brine. Fish to brine ratio was 1.5:1 (w:w). To facilitate diffusion of brine components to herring meat, each jar was gently turned around its own axis several times a day, for 4 days. After 7 days of marinating, the fillets and the brine were transferred to large funnels and left until the liquid and solid fractions were fully separated (ca. 10 min at room temperature). Both, solid and liquid fractions were weighed (with the accuracy of ± 0.1 g) and subjected to following analysis.

### Moisture, total lipids, pH, total acidity, salt and total nitrogen

The pH values of minced meat homogenized with distilled water in a ratio of 1:5 (m:m) or of brine were measured using a digital pH-meter. Contents of moisture and total lipids, and total acid value expressed as acetic acid %, as well as salt and total nitrogen contents were determined using analytical techniques according to AOAC (1990). All analyses were performed in three replications.

### Non-protein nitrogen (NPN) fractions analysis

Contents of the following compounds were determined in extracts from meat and brine at the final concentration of trichloroacetic acid at 5%: (1) non-protein nitrogen with the Kjeldahl method (AOAC 1990); (2) α-amine nitrogen with the Pope-Stevens method (1939); (3) products of protein hydrolysis: tyrosine [PHB(A)] and peptides [PHB(R)] with the modified Lowry method (Kołakowski et al. 2005); and (5) total volatile bases nitrogen (TVB-N) and trimethylamine (TMA-N) with the microdiffusion method (Conway 1947). All analyses were performed in three replications.

### Peroxide and anisidine value of lipids

The peroxide value (PV) of meat lipids was determined with the thiocyanate technique (Mihaljević et al. 1996), based on oxidation of ferrous salt with hydroperoxides and the reaction of ferric salts with potassium isothiocyanate. The red ferric complexes formed were determined spectrophotometrically. ThePV was expressed as meq O_2_/kg lipids. The anisidine value (AsV) and total oxidation value (Totox) were determined according to the ISO6885 method.

### Antioxidative activity

The antioxidative activity of fish meat and brine was determined according to Re et al. (1999) as the total antioxidative capacity of buffer (TEAC-H_2_O) and methanolic extracts (TEAC-MeOH). The DPPH˙ scavenging activity (RSA) was determined in methanolic extracts according to Brandt-Williams et al. (1995) and ferric reducing antioxidant power (FRAP) was determined in a buffer extract according to Benzie and Strain (1996). All results were expressed as Trolox equivalents (µTE) per 1 g of sample.

### Enzyme assay

General proteolytic activity was assayed against acid hemoglobin according to Anson method with modifications (Szymczak 2017). The general proteolytic activity was defined as mg tyrosine liberated per 1000 g meat or 1000 mL brine at 37 °C within 2 h (U_Hb_). The activity of D-like cathepsin was determined using 1 µM pepstatin-A (Sigma–Aldrich, Poland) and its percent inhibition was calculated. Analyses were performed in three replications.

Activities of aspartyl and cysteine peptidases were measured against Mca-GKPILFFRLK(Dnp)-r-NH2 and Z-FR-MCA (PeptaNova, Concord, CA, USA), respectively (Szymczak 2017). Fluorescence was measured with a Spectrofluorometer (Hitachi, F-7000, Tokyo, Japan) using microcuvette with excitation and emission wavelengths at 328 and 393 for aspartyl, and 322 and 460 nm for cysteine peptidases, respectively. One unit of enzyme activity (U_MCA_) was defined as 1 nmol MCA released from 1 g of meat or 1 mL of brine per minute at 37 °C. Analyses were performed in two replications.

### Sensory profiling

The samples of marinades were analyzed by sensory profiling performed by a trained sensory panel, using a five-point scale with 0.5-point accuracy (Szymczak et al. 2013). Briefly, three skinned fillets from each sample were served in porcelain trays. The assessors used water and flat bread to clean their palate between samples. A vocabulary was developed during the initial training sessions. The sensory attributes were texture, flavor, odor, and appearance. The sum of individual scores gave a total score that represented the overall sensory evaluation of the marinades.

### Texture analysis

Hardness was determined in 4 fillets from each sample with a TA-XT 2/25^®^ Texture Analyzer (Stable Micro Systems, Godalming, UK). Hardness tests included two-fold penetration of a cylindrical pin P10, with sample deformation up to 50% of its height at the speed of 5 mm s^−1^, and were conducted for each fillet separately (in 3 replications each), only in the central part of the dorsal muscle. Thier course was recorded as curves representing changes of force in time.

### Enumeration of specific microbial genera

Microbiological analyses of frozen-thawed and marinated herring meat, and brine waste before and after filtration included: total count of psychrophilic and mesophilic bacteria, yeast and molds, and lactic acid bacteria, as previously described by Szymczak et al. (2013).

### Statistical analysis

Results were analyzed statistically using one-way analysis of variance (ANOVA) with StatSoft Statistica 9.0 software (Statsoft, Tulsa, OK). The ANOVA *P* value was set at 0.05, and the differences between treatments were examined using the post hoc Tukey’s test of honestly significant differences (*P* < 0.05). Data from meat analyses were subjected to the Principal Component Analysis (PCA) based on correlations.

## Results and discussion

### Raw material: herring

The study was conducted with frozen fillets of Atlantic herring having the size of 4–8 fillets/kg that are most often used in the fish processing industry for marinating. Thawed fish was of good quality. Fish meat had a high pH value and low content of N-TVB, trimethylamine in particular (Table 1). It contained over 18% of fat and was characterized by low values of lipid oxidation indices. The antioxidative and proteolytic activities in the tissue were typical of frozen herring, i.e. the TEAC value was lower while the FRAP value was higher than in fresh one (Kołakowska and Bartosz 2011), and aspartyl peptidases were predominating (Szymczak 2017). The microbiological quality of herring was appropriate as well (Table 1). Meat contained mainly psychrophiles, which probably originated from fish catching and processing.

### Preparation and characteristics of brines

Brine left after herring marinating contained 3.8% of salt, 1.3% of acetic acid, it had pH of 4.48, and simultaneously contained high amounts of nitrogen compounds and lipids (Table 2). Such a composition facilitates rapid spoilage of brine, therefore it was immediately filtrated. Salt and acetic acid were refilled, and the brine was used for herring marinating. The brine was purified via particle- and micro-filtration. Unlike ultrafiltration, these methods are inexpensive, easy and fast to perform, and do not increase temperature of the purified solutions. Firstly, the brine was filtered through a filter with pore size of 50 µm, and then through a 0.22 µm filter. Filtration was conducted under reduced pressure to increase pressure gradient and accelerate filtration. On the industrial scale, the filtration process of brine waste may be performed by using inexpensive bags made of plastic with pore diameters ranging from 100 to even 0.1 µm. The bag filters may be mounted in stands which may be coupled in parallel, with the option of vacuum filtration enabled.

Unfiltered brine (UB) contained 775 mg of total nitrogen, of which almost 1/5 was constituted by non-protein nitrogen (Table 2). In addition, the brine contained high amounts of α-amine nitrogen as well as peptide [PHB(R)] fraction and free amino acid [PHB(A)] fraction. Many of these compounds, like free amino acids and oligopeptides, exhibited antioxidant activity, which was confirmed by results of TEAC, RSA and FRAP analyses (Table 2). Bioactive compounds of the brine include also enzymes (Szymczak 2016a, b, 2017). In the case of the marinating process, especially significant are endogenous muscle proteases active in acidic conditions. The general proteolytic activity of the brine reached 53 U_Hb_, of which 90% was due to the activity of cathepsin D. The ratio of the activities of aspartyl to cysteine peptidases was at 4:1. Apart from the aforementioned beneficial compounds, brine waste contained also some undesirable ones, like: (1) volatile ammonium bases up to 10 mg, (2) 0.43% of lipids with increased values of oxidation indices and (3) microbiological contaminants in counts ranging from 1.9 to 3.08 Log(cfu/ml) depending on bacteria group.

At the first stage of brine purification, particles of fish tissue were falling down on a 50 µm filter and formed a fouling cake layer that behaves as the second barrier to particle transport, which additionally increased the degree of BP50µ permeate purity. Results obtained demonstrate that the particle filtration had an insignificant effect on decreasing concentrations of salt, acetic acid and nitrogen fractions in the brine (Table 2). The content of total N decreased only by 3%, which indicates that most of the proteins occurred in the brine in the soluble form. The count of psychrophilic bacteria decreased as well, whereas yeast and molds were not detected in BP50 µm permeate. The particle filtration had the greatest impact on lipids’ concentration in the brine, which decreased 43-fold. In contrast, values of lipid oxidation indices were observed to increase. Other unpublished results show that the 50 µm filter often enabled complete removal of lipids from the brine. The BP50 µm brine permeate was characterized by 25% lower general proteolytic activity and activities of aspartyl and cysteine peptidases, which were probably bound by the fouling cake layer.

The prefiltration/particle-filtration stage was indispensable to remove most of the suspension and lipids from the brine, which enabled the second stage of purification, i.e. microfiltration. The direct use of the 0.22 µm filter for brine waste purification is impossible. Results obtained demonstrate that this filter had even more beneficial effect on brine parameters compared to the particle filter. The concentration of total N decreased by another 6.5%, while lipids were not detected in the BP022 µm permeate (Table 2). Worthy of notice is that the use of the 0.22 µm filter caused an increase of the proteolytic activity in the brine to the initial value measured in the UB brine. This results from damage of membranes of lysosomes present in the brine and from the release of cathepsins from them (Szymczak 2016a). Lysosomes are from 0.1 to 1 µm in size, hence the increase in the proteolytic activity after microfiltration depends mainly on the cut-off parameter of the filter and pressure during filtration. It was noticed that damages of lysosomal membranes in the filtrated brine may also be indicated by the increased concentration of the peptide fraction (Table 2). Microfiltration enabled also complete reduction of microorganisms from BP022µ permeate. Filter size of 0.2 µm corresponds to the size of the smallest bacteria. Therefore, the presence of the smallest mesophiles in the filtrated brine may be explained by the heterogeneous pore size in the applied 0.22 µm filter (Cheryan 1998). In food technology, microfiltration is classified among methods of cold sterilization and offers almost 100% effectiveness against viable cells and resting spores (Paraskeva and Graham 2005). It is very common in, e.g. the dairy industry for bacterial removal, milk tailoring, selective separation of micellar casein and milk fat, fat removal and cheese brine purification.

After filtration, salt and acetic acid were refilled in the brines. Increasing the ionic strength and decreasing the pH value of the brine caused precipitation of proteins and peptides, therefore the refilling of the above compounds was conducted after brine filtration to avoid retention of enzymes and nitrogen compounds with antioxidant properties on the filter. We presume that in the regenerated brine the precipitated proteins were re-dissolved along with increasing pH of the medium during marinating. The NPN and its fraction exhibit buffering properties, hence once acetic acid is added to the brine waste it is necessary to determine acid concentration and adjust it to the desired value.

### Marinated herring meat

Herring meat marinated in the fresh brine (FB) differed significantly from that marinated in the brine waste. Filet mass yield after marinating in FB was insignificantly lower by 0.5–0.9 percentage point (pp) than in brine permeates (Table 3). It is known that differences in pH value of the meat of the analyzed marinades by 0.1 and differences in salt concentration by 0.03–0.11 pp (Table 3) have no significant effect on mass yield of marinated herrings (Szymczak et al. 2015). Probably, the difference in mass yield results from lower water holding capacity of FB fillet meat and from greater losses of nitrogen fractions during marinating.

**Table 3:** Characteristics of Atlantic herring fillet meat after 7 days of marinating in different brines

Analysis/parameter	Unit	Fresh brine (FB)	Brine waste		
			Unfiltered (UB)	Brine permeate	
				BP50µ	BP022µ
Mass yield	%	89.6^a^	90.1^a^	90.4^a^	90.1^a^
NaCl		2.60 ± 0.02^a^	2.57 ± 0.00^a^	2.49 ± 0.01^b^	2.52 ± 0.01^b^
Acetic acid		2.00 ± 0.01^a^	2.01 ± 0.04^a^	1.87 ± 0.00^b^	1.91 ± 0.02^b^
Moisture content		59.9 ± 0.20^a^	60.8 ± 0.20	59.5 ± 0.20^a^	60.1 ± 0.10^a^
pH	Value	4.30 ± 0.002	4.40 ± 0.002^a^	4.40 ± 0.001^a^	4.42 ± 0.001
Total N	g·100 g^−1^	2.74 ± 0.09^a^	2.78 ± 0.02^a^	2.70 ± 0.03^a^	2.72 ± 0.10^a^
Non-protein N	mg·100 g^−1^	295 ± 0	365 ± 6^a^	367 ± 3^a^	365 ± 0^a^
PHB(R)		748 ± 6^a^	771 ± 55^a^	748 ± 58^a^	701 ± 64^a^
PHB(A)		113 ± 3^a^	137 ± 3^b^	138 ± 4^b^	132 ± 5^b^
α-amine N		61.6 ± 0	78.4 ± 0^a^	78.4 ± 0^a^	78.4 ± 0^a^
N-TVB	mg·100 g^−1^	6.16 ± 0.49	9.24 ± 0.28^a^	8.68 ± 0.28^a^	8.13 ± 0.16
N-TMA		0.47 ± 0.16^a^	1.21 ± 0.16^b^	0.84 ± 0.0^ab^	0.75 ± 0.16^ab^
N-NH_3_		5.69 ± 0.32	8.03 ± 0.16^a^	7.93 ± 0.43^a^	7.47 ± 0.16^a^
Lipids	%	22.8 ± 2.2^ab^	23.6 ± 1.3^a^	23.9 ± 0.2^a^	20.5 ± 1.7^b^
AsV	Value·100 g^−1^ lipids	6.86 ± 0.10	3.00 ± 0.06	1.31 ± 0.09	0.19 ± 0.11
PV	mEq O_2_·kg^−1^ lipids	7.42 ± 0.00^a^	7.70 ± 0.25^a^	6.21 ± 0.04	4.67 ± 0.15
Totox	Value	21.7 ± 0.1	18.4 ± 0.4	13.7 ± 0.2	9.5 ± 0.4
GPA	U_Hb_	121.0 ± 6.1	142.1 ± 9.9^a^	140.0 ± 7.0^a^	138.7 ± 4.2^a^
Cat D	%	68	77	82	75
Aspartyl peptidases	U_MCA_	1.54 ± 0.03	1.75 ± 0.03	1.93 ± 0.04^a^	2.04 ± 0.03^a^
Cysteine peptidases		0.24 ± 0.03	0.39 ± 0.02	0.54 ± 0.02^a^	0.66 ± 0.04^a^
TEAC-H_2_O	µTE·g^−1^	0.85 ± 0.01^a^	0.81 ± 0.23^a^	1.13 ± 0.07	0.60 ± 0.06^a^
TEAC-MeOH		2.02 ± 0.05	2.24 ± 0.06	2.63 ± 0.03^a^	2.66 ± 0.16^a^
RSA		0.100 ± 0.001	0.106 ± 0^a^	0.108 ± 0.001^a^	0.106 ± 0.002^a^
FRAP		47.0 ± 2.2	56.6 ± 2.6^a^	75.9 ± 4.3	59.2 ± 5^a^
Hardness	N	15.3 ± 1.6^a^	14.7 ± 1.6^ab^	14.8 ± 1.3^ab^	12.3 ± 1.1^b^
Overall sensory evaluation	Points	4.25 ± 0.08	4.40 ± 0.1^a^	4.45 ± 0.02^a^	4.48 ± 0.04^a^
Psychrophiles	Log(cfu·g^−1^)	2.60	3.11 ± 0.05	2.75 ± 0.23	2.09 ± 0.07
Mesophiles		0	0	0	0
Molds and yeast		1.70	3.08	0	0
LAB		0	0	0	0

^abc^Means in row without lower case letter or with different lower case letter differ significantly (*P* < 0.05); For details see “Materials and methods” section

Herring marinating in unfiltered brine (UB) caused an 8% increase in the content of total nitrogen in meat, mainly due to a significant (α = 0.05) increase (by as much as 25%) in the content of non-protein nitrogen (NPN) (Table 3). The increase in NPN content was mainly attributed to increased contents of α-amine nitrogen (27%) and free amino acid fractions (21%). Upon the use of BP50µ permeate, the content of peptide fraction did not change in herring meat, but decreased significantly by 6% upon the use of BP022µ permeate (Table 3). The increase in NPN content and in contents of its individual fractions in herrings marinated in brine waste is due to multiple phenomena. Primarily, it is an effect of reverse diffusion from brine waste to fish meat, which occurs: (1) within the first 2 days when acetic acid and salt penetrate into meat, and (2) at the second stage of ripening when herring tissue becomes more loose. The second positive phenomenon is the reduction of nitrogen losses from meat to the brine, which was described below. Probably, NPN content increase in meat was also affected by enzymatic and antioxidative phenomena.

**Fig. 1:**
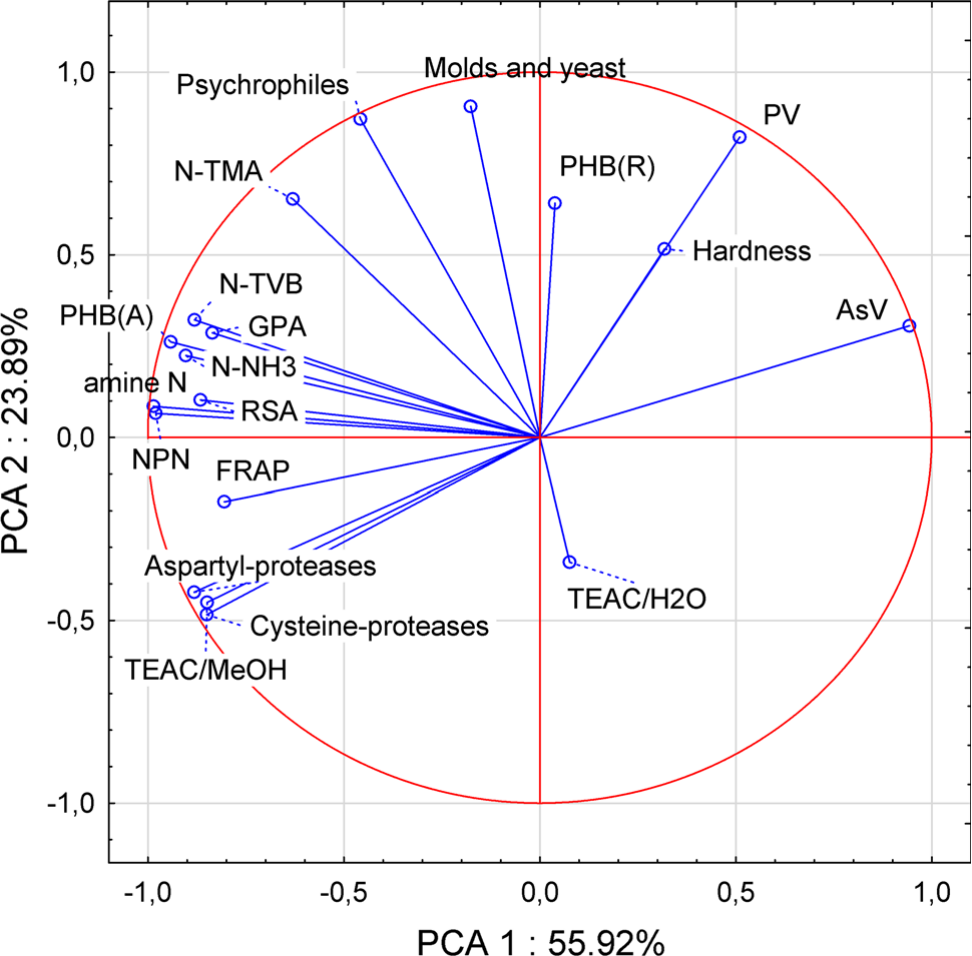
PCA biplot for parameters of herring meat marinated in fresh and waste brine. For details please see “Materials and methods” section

The general proteolytic activity (GPA) of marinated meat was the lowest when fillets were marinated in fresh brine. Marinating in brine waste increased the GPA value and content of cathepsin D by 16% (Table 3). Differences in the pH value and salt content of meat were too low to induce such a high increase in GPA (Szymczak 2017). Probably, part of proteases present in the brine waste diffused back to herring meat. Reduction of losses of proteases from meat to brine is also likely. Determination of the activity against specific substrates demonstrated that the use of brine waste, and particularly of the permeate after microfiltration, significantly enhanced activities of aspartyl and cysteine peptidases in marinated herring meat. This means that the higher proteolytic activity of meat also affected an increase in the content of NPN and its individual fractions in herring meat marinated in the brine waste. The statistical analysis confirmed a strong positive correlation between the activity of proteases and contents of NPN and its fractions (Fig. 1).

Significant was also the increase in the antioxidative activity of herring meat marinated in brine waste (Table 3). This increase was due to reverse diffusion of peptides and amino acids, oligopeptides in particular, which exhibit the highest antioxidant activity (Shihadi and Zhong 2010). Also Gringer et al. (2016) demonstrated that after the marinating process, nitrogen compounds of brines still exhibit the antioxidative activity. This observation encouraged other researchers to use the recovered fraction of peptides for the coating of fish to decrease lipid oxidation rate (Albertos et al. 2016). Results of our study indicate that the antioxidant activity TEAC-MeOH increased significantly in meat marinated in BP50µ and BP0.22µ, as a result of increased contents of amino acids (especially the hydrophobic ones) and oligopeptides in meat. The insignificant changes of TEAC-H_2_O may result from the binding of part of peptides and amino acids be free radicals present in the brine, which was reflected in reduced lipid oxidation (Table 3). The RSA is affected by contents of amino acids and small peptides with molecular weights in the range of 1400–1600 Da, including especially these containing aromatic groups, and soluble in organic solvents (Kołakowska and Bartosz 2011). The RSA was strongly correlated with contents of NPN, α-amine N and PHB(A) (Fig. 1). The highest FRAP value is typical of NPN compounds with molecular weight up to 3 kDa (Gringer et al. 2014), hence the use of brine waste allowed achieving a significantly higher ferric reducing ability of plasma in meat (Table 3). TEAC-MeOH, RSA and FRAP correlated negative with lipid oxidation indices, what indicates that the antioxidative compounds were characterized by effective activity (Fig. 1). The high contents of NPN and its fraction were the most strongly correlated with RSA and FRAP, and less strongly with TEAC-MeOH, whereas results of TEAC-H_2_O assay were weakly negatively correlated with the content of the peptide fraction (Fig. 1). In addition, herring meat marinated in UB had over 50% lower anisidine value (AsV). In turn, the use of BP50µ permeate caused a five-fold decrease in the AsV and a 16% decrease in the peroxide value (Table 3). When herrings were marinated in the lipid-free BP022µ permeate (Table 2), even a greater decrease was determined in the values of lipid oxidation indices in marinated herring meat, i.e. by 97 and 40%, respectively. It is important as fatty acid oxidation products interact also with proteins of enzymes and by this means suppress the proteolytic activity of fish (Zhang et al. 2013). The use of brine waste with a high NPN content enables reducing oxidation of herring meat lipids, which may additionally significantly extend the shelf-life and maintaining good quality of marinades.

Hardness of meat of herring fillets marinated in fresh brine reached 15.3 N (Table 3). When the fillets were marinated in UB and BP50µ permeate, meat hardness decreased only by 5%. In turn, the use of BP022µ permeate reduced its value by as much as 20%. This may indicate that the increase in the proteolytic activity had a lesser effect on meat hardness than the reduced values of lipid oxidation indices. It confirms that oxidation products are responsible for protein crosslinking and for increasing the hardness of salted herring meat (Andersen et al. 2007). These observations were confirmed in the statistical analysis which demonstrated a strong positive correlation between meat hardness and PV + AsV, and a weak negative correlation between meat hardness and GPA (Fig. 1). The lower hardness of herring meat marinated in brine waste was also demonstrated in the sensory assessment. Meat of the marinades ripening in brine waste was assessed as more ripe, juicy and sweeter. Meat of herrings ripening in BP022µ permeate was the most juicy. Also the appearance of marinated herrings depended on brine type. The overall sensory assessment of herrings marinated in FB was lower by 0.15–0.23 points compared to herrings marinated in brine waste (Table 3).

### Losses of non-protein nitrogen

Losses of non-protein nitrogen from meat to brine during marinating significantly deteriorate the quality of herring marinades (Szymczak and Kołakowski 2012). NPN losses from meat of herrings marinated in brine waste were calculated considering the initial concentration of NPN in UB, BP50µ and BP022µ. Results of these calculations demonstrated that marinating in brine waste allowed reducing NPN losses by as much as half (Table 4). These results confirmed an earlier observed phenomenon that an increase in NPN content in meat of herrings marinated in brine waste results from reverse diffusion and minimization of losses. The lesser diffusion of NPN from meat to brine waste results probably from a lower gradient of NPN concentrations between the meat and the brine. This may indicate that the use of brine waste with even higher concentration of NPN allows for even greater reduction of losses of valuable nitrogen fractions from meat to brine.

**Table 4:** Losses of non-protein nitrogen (NPN) from meat to brine during herring marinating in different brines

Sample	Losses of NPN in whole brine (g)	Losses of NPN in whole brine versus 100 g raw herring (g)	Losses of NPN in whole brine versus TN in raw herring (%)
FB	1.519	0.148	6.28
UB	0.759	0.075	3.20
BP50 µm	0.805	0.080	3.41
BP0.22 µm	0.533	0.071	3.01

*FB* fresh brine; *UB* unfiltered brine waste; *BP50µ* brine permeate 50 µm filter; *BP022µ* brine permeate 0.22 µm filter

### Safety of using brine waste

Brine waste contains many valuable substances which increase the quality of marinated herrings. However, the inappropriate reuse of brine waste may pose hazards or deteriorate the quality of meat of marinades. The brine left after fish marinating contains bacteria which originate from raw material and from cross-contamination induced by the staff and environment of a processing plant (Nędzarek et al. 2017). Despite high concentrations of acetic acid, salt and even preserving agents, bacteria proliferate abruptly in the brine as they have access to high amounts of easily-available nutrients like free amino acids. Results of our study show that the microbiological safety of brine waste allows for its microfiltration with a 0.22 µm filter. Bacterial contamination of marinades ripening in BP022µ permeate was even lower than in the marinades ripening in the fresh brine. This is, probably, due to antibacterial properties of peptides of fish origin (Halim et al. 2016), which could be confirmed by a negative correlation demonstrated between the count of psychrophilic bacteria and TEAC-H_2_O, and by a weak positive correlation shown between bacteria count and NPN content.

The content of nitrogen of volatile ammonium bases (N-TVB), as an indicator of freshness of fish products, was strongly positively correlated with bacterial count in meat of the marinades (Fig. 1). The marinades ripening in brine waste had increased values of N-TVB by 30–50%, N-TMA by 60–250% and N-NH_3_ by 30–40%. The greatest increase was noted upon the use of UB, and the least one upon the use of BP022µ permeate (Table 3). TVB easily diffuse from or to meat of marinated herrings (Szymczak and Kołakowski 2016). In spite of that, N-TVB content at 8–10 mg is several times lower than the permissible value of 35 mg (EC 2005).

Herring meat contains also heavy metals and polychlorinated biphenyls that can accumulate during the re-use of brine waste. Using microfiltration, it is possible to remove a significant portion of these impurities from various wastewater from the food industry (Nędzarek et al. 2017).

## Conclusion

The reuse of brine for marinating solves sewage problems and, additionally, increases the quality of meat of marinades. Marinades obtained by the new method have a higher content of biologically active compounds.

Beneficial is only the use of brine waste after particle-filtration, and even more beneficial—after microfiltration. Quality indices of herrings ripening in 0.22 µm permeate were even better than these of herrings ripening in fresh brine. The increased quality of meat of the marinades is due to the minimization of losses of NPN and proteases from meat to brine, and to the reverse diffusion of NPN and proteases from brine to meat.

Probably in the future the processes of fish marinating and salting will be based on the reuse of brine. Further investigations on this method of fish marinades production should lead to its successive development. This is very important because the quality of herring marinades and production cost on the industrial scale is still unsatisfactory.
